# Role of mean platelet volume in hypertriglyceridemia-induced acute pancreatitis during pregnancy

**DOI:** 10.1186/s12884-020-03295-y

**Published:** 2020-10-06

**Authors:** Longhuan Zeng, Xueying Cai, Jiayi Chen, Guangyong Jin, Yongke Zheng

**Affiliations:** Department of Intensive Care Unit, Affiliated Hangzhou First People’s Hospital, Zhejiang University School of Medicine, 261 Huansha Road, 310006 Hangzhou, Zhejiang China

**Keywords:** Hypertriglyceridemia-induced acute pancreatitis, Pregnancy, Mean platelet volume, Diagnosis, Severity

## Abstract

**Background:**

Hypertriglyceridemia-induced acute pancreatitis during pregnancy (HTG-APP) is a rare but severe disease with high maternal-fetal mortality risk, which constitutes a systemic inflammatory process accompanied by thrombosis and bleeding disorders. However, the role of mean platelet volume (MPV) in HTG-APP remains unclear.

**Methods:**

In the retrospective study, we collected 45 patients with HTG-APP as the HTG-APP group and 49 pregnant females with hypertriglyceridemia as the control group. MPV and other relevant variables at onset and remission were collected and compared.

**Results:**

MPV were significantly higher in the HTG-APP group than in the control group (*P* < 0.001), and lower in remission than on onset (*P* = 0.002). According to the severity of acute pancreatitis, all subjects were classified into mild AP (MAP), moderately severe AP (MSAP), and severe AP (SAP) groups. There was a significant difference in MPV on onset among the three groups (*P* = 0.048), and the SAP patients had the highest levels of MPV. In addition, only in the SAP group, MPV was lower in remission than on onset (*P* = 0.010). Logistic regression analyses revealed that MPV was significantly associated with SAP (odds ratio = 2.077, 95% confdence interval, 1.038–4.154; *P* = 0.039).

**Conclusions:**

These results may indicate an important role of mean platelet volume in evaluating the severity of HTG-APP.

## Background

Acute pancreatitis during pregnancy (APP) is a rare, but life-threatening disease with an estimated incidence of approximately 3–10/10,000 [1, 2]. AP can result in multiple organ dysfunction and disseminated intravascular coagulation owing to activation of the inflammatory and coagulation systems. Moreover, pregnancy-related physiologic alterations influence the diagnosis and management of many diseases. Therefore, APP can severely affect the mother and fetus and lead to a higher risk of intrauterine fetal death.

The most common cause of APP is gallstones, followed by alcohol abuse, hypertriglyceridemia, and unknown causes [3]. Non-gallstone pancreatitis is thought to be related to more complications and a poorer prognosis, such as hypertriglyceridemia-induced AP (HTG-AP) [4].

Mean platelet volume (MPV) is a blood parameter used for measuring platelet size and is accepted as a widely used indicator of thrombocytic activity. In addition, MPV has been investigated in various thrombotic and inflammatory diseases [5].

To date, there have been few studies reporting the association between MPV and AP, and the results have been inconsistent [6–9]. Furthermore, fewer reports have focused on the relationship between MPV and HTG-AP during pregnancy (HTG-APP), and the role of MPV is unclear in this population. Therefore, we designed the current study to assess the role of MPV and the potential relationship with disease severity in HTG-APP.

## Methods

### Ethical approval of the study protocol

This study complied with the Declaration of Helsinki [10] and was approved by the Ethics Committee of Hangzhou First People’s Hospital. All patients enrolled in the study submitted written informed consent.

### Patient enrollment

In this retrospective study, we collected 45 patients with HTG-APP (HTG-APP group). The patients were hospitalized in the intensive care unit (ICU) at Hangzhou First People’s Hospital (Hangzhou, China) from January 2010 to December 2018. As a control group, 49 consecutive pregnant females with hypertriglyceridemia were included. Patients who had biliary AP, idiopathic AP, alcohol intake, trauma, pre­existing chronic pancreatitis, and a history of AP were excluded from the study. This study was approved by the Ethics Committee of Hangzhou First People’s Hospital and complied with the ethical standards.

Based on the Chinese guidelines for AP and HTG-AP, patients with AP who had a triglycerides level ≥ 11.3 mmol/L (1000 mg/dl) or between 5.65 and 11.3 mmol/L (500–1000 mg/dl), but with lipemic serum after excluding gallstone, alcohol, or medication factors, were diagnosed with HTG-AP [11].

The severity of AP was classified according to the 2012 revised Atlanta Classification, as follows: mild AP (MAP), patients without organ failure and without local complications; moderately severe AP (MSAP), patients with organ failure for < 48 h or local complications; and severe AP (SAP), patients with organ failure for > 48 h [12]. All patients were categorized into the following three groups: MAP, MSAP, and SAP. Since the main purpose of this study was to distinguish SAP in the early stage of the disease, MSAP and MAP were merged with the Non­SAP group, while AP with SAP was considered the SAP group. We defined gestational age as the first (before 12 weeks), second (13–27 weeks), and third trimesters (28 weeks until delivery).

### Data collection

The first day of hospitalization in the ICU was designated as the onset, and the day that a patient was discharged from the ICU was designated as remission when the clinical symptoms and biochemical tests were normal. Clinical data, including the age, gestational age, Ranson score, medical history, complications, and treatment were collected. Laboratory indicators, including the white blood cell (WBC) count, platelet count, MPV, hematocrit, high-sensitivity C-reactive protein (hs-CRP), total bilirubin, alanine aminotransferase (ALT), aspartate aminotransferase (AST), albumin, lactate dehydrogenase (LDH), triglycerides, total cholesterol (TC), glucose, creatinine, amylase, calcium, and D-dimer, were measured and collected at the onset. Except for the control group, the platelet count, MPV, and D-dimer were collected in remission.

### Statistical analysis

The Kolmogorov-Smirnov test was utilized for estimating the compatibility of normally distributed data. Continuous data were expressed as the mean ± standard deviation or median and 25^th−^75th percentiles as appropriate. All normally-distributed data were compared using independent samples or paired Student’s t-tests. Data shown to be non-normally distributed were analyzed using the Mann-Whitney U test or the Wilcoxon rank-sum test. Multi-group comparisons were made using one-way ANOVA or the Kruskal-Wallis H test, depending on the distribution of variables. Logistic regression analysis was used to determine the relationship between MPV as well as other clinical data andHTG-APP severity. In the model, SAP is defined as dependent variable, and we entered MPV, triglyceride as well as those variables with significant differenes at a *P*-value < 0.05 and in backward wald fashion. For all tests, a two-tailed *P*-value < 0.05 was considered statistically significant. All calculations were performed using SPSS (version 16.0 for Windows; IBM, Armonk, NY, USA).

## Results

Forty-five patients with HTG-APP and 49 control subjects were enrolled in the present study. Of the 45 pregnant women with HTG-AP, the mean gestational age was 31.22 ± 6.65 weeks, with most of the cases occurring in the third trimester (69%). MAP was diagnosed in 38% (*n* = 17) of the patients, MASP in 29% (*n* = 13), and SAP in 33% (*n* = 15). The clinical characteristics, complications, and treatment of study participants are summarized in Table 1. One patient with SAP died during the ICU stay.

**Table 1:** Baseline characteristics in patients with HTG-APP (*n* = 45)

Variables		Value
During in ICU (days)		5.36 ± 4.18
Ranson score, median (range)		1 (0–3)
**Trimester** (n, %)		
First		3 (6.7%)
Second		11 (24.4%)
Third		31 (68.9%)
**Comorbidity** (n, %)		
Fatty liver		11 (24.4%)
Gestational diabetes mellitus		5 (11.1%)
Pregnancy-induced hypertension		2 (4.4%)
**Local complications** (n, %)		
Acute necrotic collections		6 (13.3%)
Walled-off pancreatic necrosis		14 (31.1%)
Acute peripancreatic fluid collections		1 (2.2%)
Pancreatic pseudocyst		8 (17.8%)
**Systemic complications** (n, %)		
Circulatory failure		5 (11.1%)
Respiratory failure		12 (26.7%)
Renal failure		4 (8.9%)
Ketoacidosis		8 (17.8%)
**Mortality** (n, %)		
Mother		1 (2.2%)
Foetus		6 (13.3%)
**Treatment** (n, %)		
Plasma exchange		32 (71.1%)
Intubation		16 (35.6%)
Abdominal paracentesis drainage		14 (31.1%)
Conservative treatment		11 (24.4%)

Table 2 shows that there were no significant differences in age, gestational age, platelet count, ALT, AST, total bilirubin, and creatinine between the HTG-APP and control groups (all *P* > 0.05).

**Table 2:** Demographic characteristics and laboratory values of the patients and controls

Variables	HTG-APP (*n* = 45)	Control (*n* = 49)	P
Age (years)	29.36 ± 4.75	28.61 ± 4.77	0.451
Gestational age (weeks)	31.22 ± 6.65	29.92 ± 9.06	0.432
During in hospital (days)	21.30 ± 14.53^*^	6.41 ± 5.68	< 0.001
WBC (× 10^9^/L)	15.26 ± 4.82^*^	9.07 ± 2.94	< 0.001
Hematocrit (%)	31.78 ± 5.18^*^	34.57 ± 4.29	0.005
Platelet (× 10^9^/L)	189.98 ± 72.23	203.45 ± 56.83	0.316
MPV (fL)	11.29 ± 1.47^*^	10.01 ± 1.54	< 0.001
HS-CRP (mg/L)	111.00 (62.00-160.00) ^*^	5.00 (3.00-11.50)	< 0.001
ALT (U/L)	18.00 (12.00-26.57)	19.00 (12.00-33.50)	0.279
AST (U/L)	19.00 (19.00-28.50)	23.00 (17.00-32.36)	0.136
Total bilirubin (µmol/L)	11.00 (7.50–16.70)	11.00 (8.00-16.15)	1.000
Albumin (g/L)	32.46 ± 7.90	33.53 ± 4.19	0.410
Calcium (mmol/L)	1.89 ± 0.38^*^	2.28 ± 0.55	< 0.001
Creatinine (µmol/L)	60.13 ± 31.73	56.98 ± 12.81	0.523
Glucose (mmol/L)	7.72 ± 4.45^*^	5.11 ± 3.30	< 0.001
Total cholesterol (mmol/L)	22.81 (13.52–37.81) ^*^	6.09 (4.91–8.55)	< 0.001
Triglyceride (mmol/L)	28.00 (17.56–56.65) ^*^	5.86 (5.35–8.26)	< 0.001
HDL-c (mmol/L)	1.93 (1.24–3.36) ^*^	1.66 (1.35–1.83)	0.034
LDL-c (mmol/L)	5.40 (3.20–9.76) ^*^	2.92 (1.99–3.68)	< 0.001
LDH (U/L)	260.00 (189.00-398.50) ^*^	173.00 (148.00-206.50)	< 0.001
Serum amylase (U/L)	224.00 (157.00-656.00) ^*^	78.00 (60.00-140.50)	< 0.001
D-dimer (µg/L)	2846.67 (1916.11–4355.00) ^*^	1857.97 (1100.00-2182.00)	< 0.001

*P < 0.05, HTG-APP versus control

Patients with HTG-APP had a significantly higher length of hospital stay than the control group (*P* < 0.001). The WBC (*P* < 0.001), hs-CRP (*P* < 0.001), glucose (*P* < 0.001), TC (*P* < 0.001), triglycerides (*P* < 0.001), HDL-C (*P *= 0.034), LDL-C (*P* < 0.001), LDH (*P* < 0.001), amylase (*P* < 0.001), and D-dimer (*P* < 0.001) levels were significantly higher in the HTG-APP patients than the control group, whereas serum calcium (*P* < 0.001) and hematocrit (*P* = 0.005) levels in the HTG-APP group were significantly lower than those in the control group (Table 2).

**Fig. 1:**
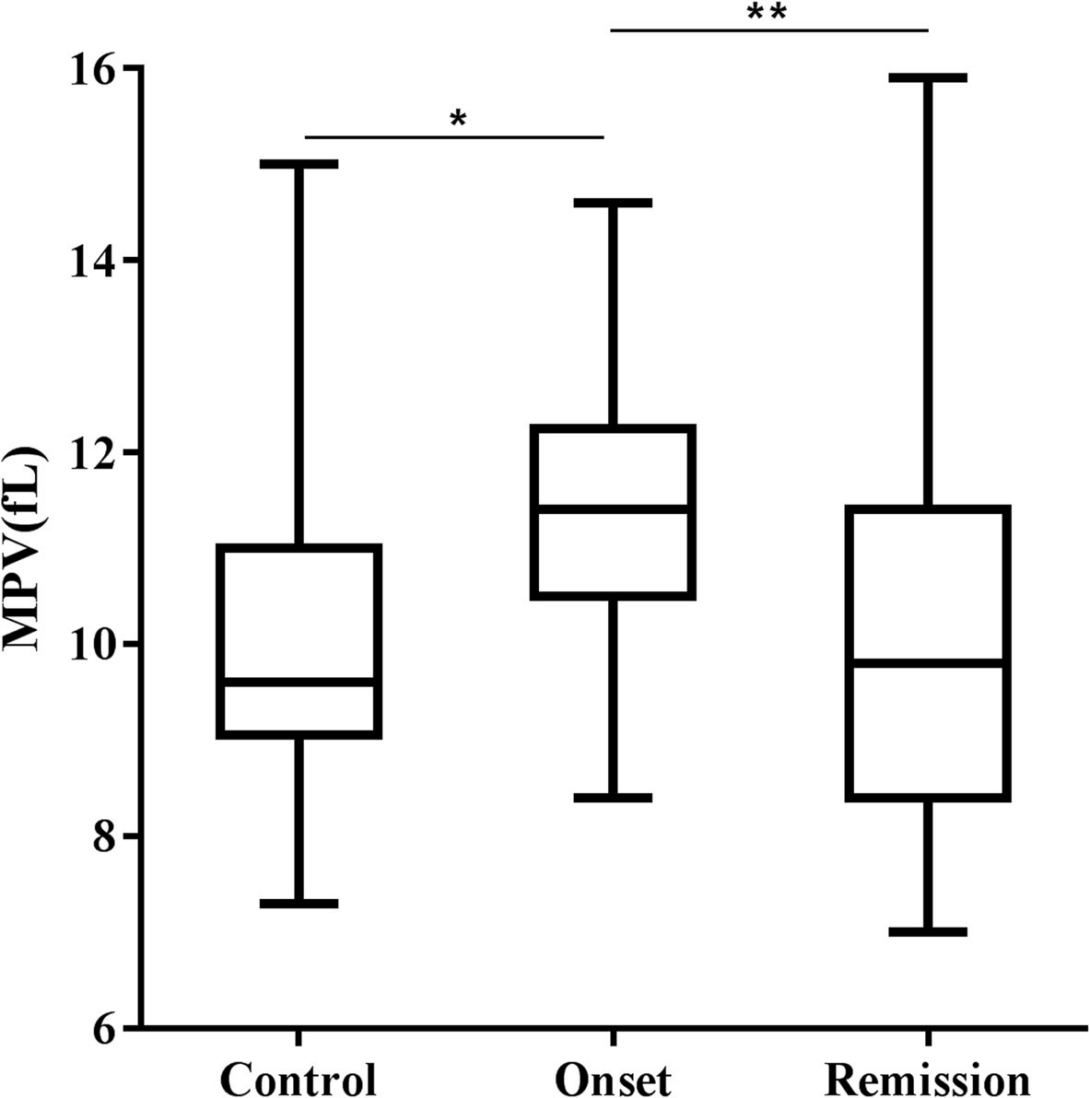
MPV levels of the patients with HTG-APP(at onset and remission) and healthy controls. *P < 0.05, Onset versus Control; **P < 0.05, Onset versus Remission

A statistically significant increase in MPV levels was observed in patients with HTG-APP compared with the control group (11.29 ± 1.47 vs. 10.01 ± 1.54, *P* < 0.001, Table 2). Figure 1 shows that the mean MPV values of HTG-APP patients at onset and in remission compared with controls.

**Fig. 2:**
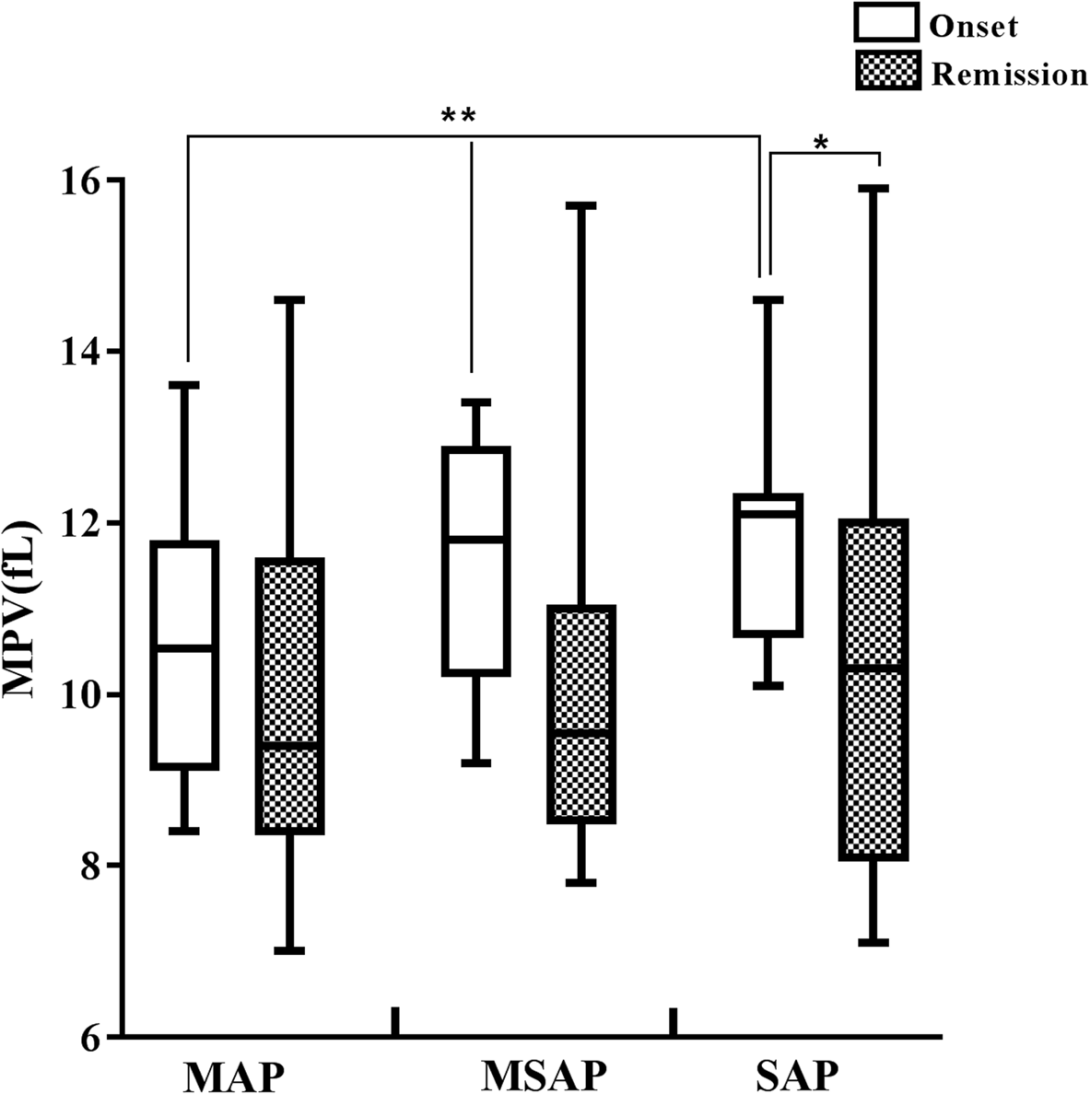
MPV levels of the patients with MAP, MSAP and SAP. *P < 0.05, Onset versus Remission.; **P < 0.05, Onset versus Remission

All HTG-APP patients were classified into SAP and Non-SAP groups. The MPV (*P* = 0.039), HS-CRP (*P* = 0.003), AST (*P* = 0.030), glucose (*P* = 0.004), creatinine (*P* = 0.005), and total cholesterol (*P* = 0.025) levels were significantly higher in the SAP group than the Non-SAP group (Table 3). The patients at onset had higher mean MPV levels than patients in remission (11.29 ± 1.47 fL vs. 10.10 ± 2.03 fL, *P* = 0.002, Table 4). Further analysis revealed a significant difference in MPV at onset among the three groups (10.64 ± 1.56 fL, 11.48 ± 1.42 fL vs. 11.88 ± 1.19 fL, *P* = 0.048), with the highest levels detected in the SAP patients (11.88 ± 1.19 fL, Table 4; Fig. 2). Differences in MPV in remission were not statistically significant among the three groups (10.00 ± 2.03 fL, 10.16 ± 2.09 fL vs. 10.16 ± 2.11 fL, *P* = 0.976, Table 4). There was also a significant difference between onset and remission in the SAP group (*P* = 0.010), but no significant differences were shown in the MAP and MSAP groups (all *P* > 0.05, Table 4). In bivariate logistic regression analysis, the MPV level was independent associated with SAP (odds ratio [OR] = 2.077, 95% confdence interval [CI], 1.038–4.154; *P* = 0.039, Table 5).

**Table 3:** Clinical Characterization of the patients with SAP and Non-SAP

Variables	SAP (*n* = 15)	Non-SAP (*n* = 30)	P
Age (years)	29.20 ± 4.46	29.43 ± 4.97	0.879
Gestational age (weeks)	33.47 ± 3.83	30.10 ± 7.47	0.110
During in hospital (days)	25.64 ± 18.98	19.27 ± 11.76	0.178
WBC (× 10^9^/L)	16.48 ± 6.44	13.29 ± 4.88	0.070
Hematocrit (%)	32.17 ± 7.46	31.59 ± 3.70	0.729
Platelet (× 10^9^/L)	181.53 ± 85.75	194.20 ± 65.65	0.585
MPV (fL)	11.88 ± 1.19^*^	10.95 ± 1.47	0.039
HS-CRP (mg/L)	151.00 (115.00-189.00)^*^	81.00 (39.25-144.25)	0.003
ALT (U/L)	16.00 (10.00–21.00)	20.00 (12.00-27.13)	0.469
AST (U/L)	27.00 (20.00–37.00)^*^	17.78 (17.00–22.00)	0.030
Total bilirubin (µmol/L)	11.00 (7.80–16.50)	10.95 (7.00-16.83)	0.847
Albumin (g/L)	29.76 ± 5.72	33.78 ± 7.71	0.081
Calcium (mmol/L)	1.78 ± 0.43	1.95 ± 0.34	0.150
Creatinine (µmol/L)	70.27 ± 20.19^*^	52.63 ± 18.15	0.005
Glucose (mmol/L)	8.69 ± 3.04^*^	6.14 ± 2.49	0.004
Total cholesterol (mmol/L)	35.37 (20.29–49.50)^*^	20.21 (11.30-33.05)	0.025
Triglyceride (mmol/L)	35.16 (18.43–62.80)	24.08 (16.86–45.57)	0.289
HDL-c (mmol/L)	3.00 (1.47–3.60)	1.85 (1.14–2.71)	0.198
LDL-c (mmol/L)	5.77 (3.64–8.30)	4.85 (2.91–10.24)	0.914
LDH (U/L)	238.00 (213.00-277.00)^*^	205.50 (184.50–248.00)	0.053
Serum amylase (U/L)	283.00 (107.00-723.00)	219.00 (160.00-486.50)	0.962
D-dimer (µg/L)	4200.00 (2340.00-5720.00)	2823.34 (1607.50-3717.50)	0.094

*P < 0.05, SAP versus Non-SAP

**Table 4:** MPV on onset and in remission in MAP, MASP and SAP group

Variable Groups		Onset	Remission	P (onset and remission)
MPV	Total (*n* = 45)	11.29 ± 1.47^*^	10.10 ± 2.03	0 002
	MAP (*n* = 17)	10.64 ± 1.56	10.00 ± 2.03	0.311
	MASP (*n* = 13)	11.48 ± 1.42	10.16 ± 2.09	0.076
	SAP (*n* = 15)	11.88 ± 1.19^*§^	10.16 ± 2.11	0.010
	P (MAP, MASP and SAP)	0.048	0.976	

*P < 0.05, Onset versus Remission

^§^*P* < 0.05, SAP, MASP versus MAP

**Table 5:** Bivariate logistic regression analyses of MPV, triglyceride as well as other clinical data and SAP in patients with HTG-APP

Variable	OR (95% CI)	P
MPV	2.077 (1.038–4.154)	0.039
Glucose	1.406 (1.035–1.908)	0.029
HS-CRP	1.000 (0.987–1.014)	0.987
Triglyceride	1.023 (1.000-1.047)	0.050
AST	1.044 (0.981–1.112)	0.176
Total cholesterol	1.004 (0.952–1.059)	0.882

## Discussion

HTG-APP is a rare but severe disease with high maternal-fetal mortality risks, which presents as a systemic inflammatory process that is often accompanied by thrombosis and bleeding disorders. Normally, triglyceride levels rise gradually during pregnancy and reach a peak in the third trimester of gestation to 2-to-4-fold over pre-pregnancy levels. In addition, high levels of estrogen related to pregnancy can reduce the activity of lipoprotein lipase and lead to hypertriglyceridemia [13] There is an increased risk for AP when serum triglyceride levels are > 10 g/L (11.3 mmol/L) [14], which is why most cases of HTG-AP occurred during the third trimester of gestation.

Currently, there are no standardized guidelines for clinicians regarding hypertriglyceridemia-induced AP during pregnancy. Moreover, the main diagnostic methods rely on clinical experience, and the existing serum biomarkers are helpful in assessing AP severity. Therefore, early, alternative, and easily applicable markers are needed.

It has been reported that platelet activation plays an important role in the development and evolution of AP [15]. MPV is a simple parameter showing platelet function and activation that can be measured by complete blood count analysis at no additional cost.

Abnormal MPV has been correlated with thrombotic and inflammatory conditions, such as myocardial infarction, acute cerebral ischemia, inflammatory bowel disease, rheumatoid arthritis, and familial Mediterranean fever [16–20]; however, the relationship between MPV and AP has not been fully clarified, especially hypertriglyceridemia-induced SAP during pregnancy.

Our finding showed that MPV levels were increased in patients with HTG-APP compared with controls. Furthermore, MPV levels were increased with the severity of HTG-AP at onset and decreased during remission.

To date, several previous studies have investigated the relationship between AP and MPV, but the results were conflicting. Beyazit et al. [21] examined the role of MPV in AP and described a significant decrease in MPV in patients with AP compared with healthy subjects. Erbis et al. [9] in a study involving 76 patients with pancreatitis showed that patients with pancreatitis had lower levels in MPV, and the mean MPV values were lower in AP patients (7.2 ± 0.52 fL) than in AP (7.9 ± 0.53 fL). Another contemporaneous study reported that MPV was significantly lower on admission than during remission in biliary and non-biliary AP patients [9]. Recently, Lei et al. [22] also reported that MPV levels were significantly lower in the AP group than the control group, and MPV presented an upward trend during the first week after admission in all AP patients.

Although the previous results suggested low MPV levels in AP, these studies excluded patients who had pregnancy or hyperlipidemia. In comparison, we focused on patients with hyperlipidemia-induced AP in pregnancy. It is probable that the disparity in the studies may be explained by differences in the study population and design. Thus, we should pay more attention to further studies.

The exact reason for increased MPV in hyperlipidemia-induced SAP during pregnancy remains unknown, but it has been speculated that platelets not only control thrombosis and hemostasis, but also regulate inflammatory conditions. There is a stimulation of megakaryopoiesis in the early stages of the inflammatory process associated with hyperlipidemia-induced SAP, which produces a large amount of young and large platelets with high procoagulation potential [23]. Osada et al. [6] observed a significant increase in the number of large platelets in the AP groups; the median and mean MPV remained at high levels during the acute phase in the mild and severe AP groups. Moreover, the MPV levels showed a downward trend in remission phase in patients with SAP, which supported our results.

In addition, MPV increases during pregancy as a result of hormonal and metabolic changes [24]. Meanwhile, thrombocytopenia is not only a common finding during pregnancy, but also frequent at the onset of AP [25]. Thrombocytopenia can lead to enhanced thrombopoiesis, which increases the quantity of highly reactive large-sized platelets [5]. Thus, these findings may partly explain why there were high MPV levels in our study patients. Akbal et al. [7] also detected higher MPV levels in patients with AP at the time of admission than controls, which was consistent with our results. In addition, our data also showed that the D-dimer was elevated in patients with HTG-APP, which were in agreement with the alterations in MPV. Together, these studies suggest that an elevated MPV facilitates platelet adhesiveness and aggregation, which may lead to a high prothrombotic potential and impairment of pancreatic microcirculation in hyperlipidemia-induced SAP during pregnancy.

There were some limitations to the present study. First, the study population was relatively small. The current study had a very limited number of patients because of the rarity of HTG-APP, which could influence the validity of some statistical models, such as regression analysis. Second, owing to ethical reasons, we could not assess the clinical benefits of antiplatelet therapy in this population. It was also difficult to adjust adequately for measurement of patient adherence. Third, this was a single-center, retrospective cohort study; therefore, the results we observed cannot be evaluated definitively. Further multi-center, large-scale, prospective studies are needed to verify the findings of the present study. Although our results may be limited in terms of the sample size and study design, the present study still offers implications for the diagnosis and management of HTG-APP. Given the specific population, we believe our findings may provide new evidence for further studies with larger sample sizes.

## Conclusions

In conclusion, our study demonstrated that the MPV levels were higher in patients with HTG-APP than the control group. In addition, the highest MPV was detected on onset in patients with hypertriglyceridemia-induced SAP during pregnancy. Furthermore, MPV levels decreased in remission in such patients. Thus, MPV could play an important role in the early diagnosis of HTG-APP and may be helpful for evaluating the severity of HTG-APP; however, additional large-scale, prospective studies are required to validate our findings.

## Data Availability

The datasets used and analyzed in the current study are available from the corresponding author on reasonable request.

## Abbreviations

ALT: Alanine aminotransferase
AST: Aspartate aminotransferase
HTG-APP: Hypertriglyceridemia-induced acute pancreatitis during pregnancy
HS-CRP: High-sensitivity C-reactive protein
HDL-c: High-density lipoprotein cholesterol
ICU: Intensive care unit
LDL-c: Low-density lipoprotein cholesterol
LDH: Lactate dehydrogenase
MPV: Mean platelet volume
MAP: Mild acute pancreatitis
MASP: Moderately severe acute pancreatitis
SAP: Severe acute pancreatitis
WBC: White blood cells
OR: Odds ratio
CI: Confidence interval
